# Prefrontal Dopaminergic Mechanisms of Extinction in Adolescence Compared to Adulthood in Rats

**DOI:** 10.3389/fnbeh.2017.00032

**Published:** 2017-02-22

**Authors:** Isabel C. Zbukvic, Chun Hui J. Park, Despina E. Ganella, Andrew J. Lawrence, Jee Hyun Kim

**Affiliations:** ^1^Developmental Psychobiology Laboratory, Behavioral Neuroscience Division, The Florey Institute of Neuroscience and Mental HealthParkville, VIC, Australia; ^2^Developmental Psychobiology Laboratory, The Florey Department of Neuroscience and Mental Health, University of MelbourneParkville, VIC, Australia

**Keywords:** extinction, fear, adolescence, dopamine, prefrontal cortex, conditioning

## Abstract

Adolescents with anxiety disorders attain poorer outcomes following extinction-based treatment compared to adults. Extinction deficit during adolescence has been identified to involve immaturity in the medial prefrontal cortex (mPFC). Findings from adult rodents suggest extinction involves dopamine signaling in the mPFC. This system changes dramatically during adolescence, but its role in adolescent extinction is unknown. Therefore, we investigated the role of prefrontal dopamine in extinction using Pavlovian fear conditioning in adolescent and adult rats. Using quantitative PCR (qPCR) analyses, we measured changes in dopamine receptor gene expression in the mPFC before and after extinction. We then enhanced dopamine 1 receptor (D1R) or dopamine 2 receptor (D2R) signaling in the infralimbic cortex (IL) of the mPFC using agonists at the time of extinction. Adolescent rats displayed a deficit in extinction retention compared to adults. Extinction induced a reduction in D1R compared to D2R gene expression in adolescent rats, whereas an increase of D1R compared to D2R gene expression was observed in adult rats. Acutely enhancing IL D1R signaling using SKF-81297 had no effect on extinction at either age. In contrast, acutely enhancing IL D2R signaling with quinpirole significantly enhanced long-term extinction in adolescents, and impaired within-session extinction in adults. Our results suggest a dissociated role for prefrontal dopamine in fear extinction during adolescence compared to adulthood. Findings highlight the dopamine system as a potential pharmacological target to improve extinction-based treatments for adolescents.

## Introduction

Anxiety disorders are the most frequent mental illness experienced by adolescents worldwide (Polanczyk et al., 2015). Exposure therapy for anxiety is based on the principle of extinction, in which fear to a stimulus can be reduced by repeated presentations of that stimulus without an aversive outcome. Adolescents attain poorer outcomes following exposure therapy for anxiety compared with children (Southam-Gerow et al., 2001; Bodden et al., 2008). Consistent with this, studies in humans and rodents showed that extinction is impaired in adolescents compared to adults and juveniles/children (Kim et al., 2011; Pattwell et al., 2012; Baker and Richardson, 2015). Specifically, extinction memory is “forgotten” in adolescent rodents that show high freezing when tested in the same context as extinction, compared to adult rodents. It has therefore been suggested that adolescent resistance to exposure-based therapies relates to deficits in extinction learning at this age (Hartley and Casey, 2013; Kim and Ganella, 2015).

The prefrontal cortex (PFC) is a critical neural region underlying extinction deficits in adolescent rodents (Kim et al., 2011; Pattwell et al., 2012; Baker and Richardson, 2015). This is not surprising, as the PFC, especially the infralimbic cortex (IL), has repeatedly been identified to be particularly important for the recall of extinction across different learning paradigms and different ages (Quirk et al., 2000; Laurent and Westbrook, 2008; Peters et al., 2009; Mueller et al., 2010; Orsini et al., 2011; Sierra-Mercado et al., 2011; Gass and Chandler, 2013; Abraham et al., 2014). To further understand adolescent vulnerability to anxiety disorders, we aim to explore the role of prefrontal dopamine 1 receptor (D1R) and dopamine 2 receptor (D2R) in fear extinction during adolescence compared to adulthood. Dopamine signaling displays a unique maturation profile during adolescence, over and above many other neurotransmitter systems in the PFC (O’Donnell, 2010; Wahlstrom et al., 2010; Kim et al., 2016). The density of dopaminergic fiber infiltration of the PFC increases throughout adolescence until early adulthood in rodents (Kalsbeek et al., 1988) and non-human primates (Rosenberg and Lewis, 1995). Dopamine synthesis also peaks in the PFC during adolescence (Andersen et al., 1997), along with dopamine receptor density in the PFC (Tarazi and Baldessarini, 2000). Importantly, age-related discontinuities in the function of the dopamine system have been suggested to underlie the adolescent behavioral phenotype observed across human and non-human mammals (Laviola et al., 2003). This includes differences in impulsivity and cue reactivity, behavioral responses critically implicated in extinction learning (Laviola et al., 2003; Pattwell et al., 2013).

Dopamine exerts its effects via five distinct receptors, which are subdivided into two families: D1-like and D2-like receptors (Andersen et al., 1990). The D1-like subfamily comprises D1R and D5R, and the D2-like includes D2R, D3R and D4R (Missale et al., 1998). The most abundant dopamine receptor subtypes in the central nervous system are D1R and D2R (Jaber et al., 1996), with both showing expression in the medial prefrontal cortex (mPFC; Vincent et al., 1993). As members of different subfamilies, D1R and D2R show distinct profiles in terms of downstream signal transduction and physiological effects (Jackson and Westlind-Danielsson, 1994; Beaulieu and Gainetdinov, 2011).

Findings in adult rats show complex involvement of prefrontal D1R and D2R signaling in fear extinction. For example, infusion of the D1R antagonist SCH-23390 into the IL of PFC impairs long-term fear extinction (Hikind and Maroun, 2008). Consistent with this, transgenic mice lacking D1R show normal fear conditioning but delayed extinction up to 90 days post-conditioning (El-Ghundi et al., 2001). By comparison, the role of D2R signaling in extinction is less clear. For instance, one study showed that pre-extinction systemic treatment with the D2R agonist quinpirole blocked extinction of conditioned fear (Nader and LeDoux, 1999), while another showed largely no effect across a range of doses (0.25, 0.5, 2.0 mg/kg), though one dose (1.0 mg/kg) impaired long-term extinction (Ponnusamy et al., 2005). However, pre-extinction systemic or intracerebroventricular (i.c.v.) injection of the D2R antagonist haloperidol has also been found to increase conditioned stimulus (CS)-elicited freezing during extinction and at test the next day (Holtzman-Assif et al., 2010). However, systemic D2R antagonism with sulpiride facilitates extinction both within-session and at test the next day (Ponnusamy et al., 2005), while one known study investigating intra-IL D2R antagonism using raclopride found impaired long-term fear extinction in adult rats the next day (Mueller et al., 2010).

Interestingly, findings on the expression of D1R and D2R in the PFC across adolescence are also varied. For example, D1R and D2R density in the PFC has been reported to be high at postnatal day (P) 40, then decline into adulthood across P60, P80, P100 and P120, with D1R declining more dramatically compared to D2R (Andersen et al., 2000). This is consistent with positron emission tomography (PET) findings in humans age 10–30 years, which show that D1R binding in the PFC decreases from adolescence into adulthood (Jucaite et al., 2010). Another human study reports a peak in D1R gene expression during adolescence compared to infancy and adulthood, with no changes in D2R gene expression across those ages (Weickert et al., 2007). In contrast, studies have also found no change in D1R gene expression or binding in PFC from P21 to P60 in rodents (Leslie et al., 1991; Tarazi et al., 1999; Tarazi and Baldessarini, 2000; Araki et al., 2007). A human study found no significant difference in PFC D1R gene expression between adolescents and adults (Rothmond et al., 2012). A study that used microarrays showed no changes relating to dopamine signaling, including D1R and D2R expression, across subjects aged 0–49 years (Harris et al., 2009). Taken together, further investigation into the changes in PFC D1R and D2R expression in adolescence and adulthood appear warranted, especially in light of the emerging role of PFC dopamine signaling in fear extinction.

To investigate the role of prefrontal dopamine signaling in adolescent vs. adult extinction, we first examined fear conditioning and extinction in adolescent and adult rats. Using real-time quantitative PCR (qPCR) analyses, we then measured extinction-related changes in mPFC D1R and D2R gene expression in adolescent and adult rats. Building on these results, we then acutely enhanced D1R or D2R signaling in IL during extinction using SKF-81297 or quinpirole, respectively. Dopamine receptor agonists were chosen so that parallels can be made to existing FDA-approved dopamine receptor agonists, which are more readily administered compared to dopamine receptor antagonists in adolescent humans (Kirino, 2012; Kim and Lawrence, 2014). Thus, results from the present chapter have strong translational potential for improving extinction-based treatments for anxiety, as well as adding to literature on the mechanisms of extinction across development.

## Materials and Methods

### Animals

Male Sprague-Dawley rats (*N* = 142) were bred in-house at the Florey Institute of Neuroscience and Mental Health. Rats were housed 2–4 per cage in individually ventilated cages, maintained on a 12-h light/dark cycle (lights on at 7 a.m.) with food and water available *ad libitum*. Rats were handled daily for 3 days prior to the commencement of behavioral experiments. Rats were P(postnatal day)35 (±1) or P88 (±1) on extinction day (those ages fall within adolescence and adulthood, respectively, Madsen and Kim, 2016). All procedures were approved by the Florey Animal Ethics Committee and performed in accordance with the guidelines of the National Health and Medical Research Council Code of Practice for the Care and Use of Animals for Experimental Purposes in Australia.

### Surgery

For intracranial infusion experiments, a double guide cannula (26 gauge, PlasticsOne) bilaterally targeting the IL (AP, +3.0 mm; ML ± 0.6 mm; DV −4.6 mm [age P88] or −4.2 mm [age P35]) was implanted stereotaxically (David Kopf Instruments, Tujunga, CA, USA). These coordinates were identified by pilot surgeries involving ink microinfusions using the rat brain atlas (Paxinos and Watson, 1998). Rats were anesthetized with isoflurane (2%–5% v/v) vaporized with oxygen and injected with meloxicam (3 mg/kg, i.p.). The cannula was secured to the skull using dental cement (Vertex, MA, USA) combined with anchoring screws (PlasticsOne, Roanoke, VA, USA). Obturators extending 1 mm below the guide cannula were inserted and covered with a metal cap. Rats received antibiotic (Baytril, Bayer Corporation; 10 mg/kg, i.p.) daily for 3 days following surgery. Obturators were checked and rats were weighed daily for 3–5 days after surgery until behavioral experimentation.

### Drugs

The bilateral infusion (0.5 μL/hemisphere) consisted of either vehicle (saline), SKF-81297 (dissolved in saline; 0.1 μg/hemisphere; Sapphire Bioscience, Redfern, NSW, Australia), or quinpirole (dissolved in saline; 1 μg/hemisphere; Tocris, UK) into the IL over 2 min. These doses were chosen based on previous studies that showed consistent effects specifically with these doses (Floresco and Phillips, 2001; Floresco et al., 2006; Lauzon et al., 2009; Zbukvic et al., 2016). The infusion cannula extended 1 mm below the guide cannula, and remained in place for 2 min following the infusion, and then rats underwent extinction. At the end of experimentation, cannula placements were validated by an experimenter who was blind to subject treatment. To visualize cannula placement, fresh frozen brains were sectioned and stained with cresyl violet (Kim et al., 2009).

### Procedures

All behavioral sessions used standard fear conditioning chambers (31.8 × 25.4 × 26.7 cm, Med Associates, St. Albans City, VT, USA), using previously published protocol (Ganella et al., 2016). A grid floor consisting of 4.8 mm stainless steel rods set 16 mm apart allowed delivery of an electric footshock, which served as the unconditioned stimulus (US). A speaker positioned in one wall of each chamber was used to produce a tone (5000 Hz, 80 dB), which served as the the conditioned stimulus (CS). Chambers were housed in cabinets insulated with acoustical soundproof foam to minimize external noise. A ventilation fan in each cabinet produced low-level constant background noise. Chambers contained a near infra-red (NIR) fear conditioning system and a monochrome video camera equipped with 8.0 mm lens and NIR pass filter was attached to the inside of each cubicle to record behavior. Freezing behavior was quantified using VideoFreeze software (Med Associates, St. Albans City, VT, USA), which shows high concordance with manual scoring as previously described (Ganella et al., 2016). Fear was measured by levels of freezing behavior, defined as a motion threshold of less than 50 pixels for a minimum of 1 s duration. All CS and US presentations were controlled and recorded by VideoFreeze software (Med Associates, St. Albans City, VT, USA).

Two separate rooms representing two different experimental contexts housed four conditioning chambers each, to administer extinction and test in a different context to conditioning as described previously in other studies examining fear extinction during adolescence (McCallum et al., 2010; Kim et al., 2011). This design is widely employed across fear extinction studies, to isolate extinction learning of the conditioned cue separate from the conditioned context (Mueller et al., 2009; Holmes and Quirk, 2010; Ganella et al., 2016).

In one context, the back wall of the chambers was covered with a plastic spot-patterned cover and a tray containing woodchip bedding was located underneath the grid floor. In this context, chambers were cleaned with eucalyptus-scented disinfectant before each session and a white houselight remained on in each chamber for the duration of all sessions. In the other context, chambers were fitted with a curved white wall that covered the sides and back walls of the chamber, trays beneath the grid floor contained paper towel, and houselights were off for the duration of all sessions and a red light was on in the room. Chambers in this context were cleaned with ethanol (80% v/v in water) before each session. The two contexts served as conditioning or extinction/test contexts in a counterbalanced manner.

#### Conditioning

On day 1 of behavioral experimentation, rats were placed in the chambers and their baseline level of freezing was recorded for 2 min. The CS tone (80 dB) was then presented for 10 s and co-terminated with a 1 s footshock (0.6 mA). There were three CS-US pairings and the inter-trial interval (ITI) between each pairing was between 85–135 s. Following the last CS-US pairing, rats remained in the chambers for 2 min before returning to their home cages.

#### Extinction

On day 2, rats received extinction in the context different to that in which conditioning took place. Baseline freezing was measured for the first 2 min, followed by 30 CS alone trials with a 10 s ITI.

#### Test

On day 3, rats were tested in the same context as extinction. Baseline freezing was measured for the first minute, followed by a 2 min presentation of the CS alone. Rats remained in the chambers for 1 min before returning to their home cages.

### Gene Expression Analysis

For the gene expression experiment the Pre-extinction group did not receive extinction but were handled for 2 min by the experimenter. The Post-extinction group underwent extinction as described. Rats were deeply anesthetized by sodium pentobarbitone injection (100 mg/kg, i.p.) 2 h following handling (Pre-extinction) or extinction (Post-extinction). Previously published protocol was used to measure mRNA levels (Chen et al., 2016). Specifically, brains were rapidly removed and sectioned using a brain matrix (*World Precision Instruments*, Sarasota, FL, USA) under RNase-free conditions. The mPFC was micro-dissected (Figure 2C), and tissue was snap frozen over liquid nitrogen then stored at −80°C.

Total RNA was extracted from the mPFC from both hemispheres using an RNeasy Mini kit (Qiagen, Malvern East, VIC, Australia), then reverse transcribed into cDNA using TaqMan Reverse Transcription reagents as per the manufacturer’s protocol (Applied Biosystems). Gene expression was analyzed by qPCR using SYBR Green Mastermix (Applied Biosystems) on a ViiA^TM^ 7 Real-Time PCR System (ThermoFisher Scientific). Three housekeeping genes (*Actb*, *Gapdh* and *Hprt1*) were assessed for stability in adult and adolescent mPFC. *Hprt1* was the least variable between groups and was used for all subsequent analyses. Primers were designed using Primer3 (Rozen and Skaletsky, 2000) as follows:

*Hprt1*   forward   5′-CTGGTGAAAAGGACCTCTCG-3′; *Hprt1*   reverse   5′-TCCACTTTCGCTGATGACAC-3′, *Drd1*   forward   5′-CCTTCGATGTGTTTGTGTGG-3′, *Drd1*   reverse   5′-GGGCAGAGTCTGTAGCATCC-3′; *Drd2*   forward   5′-TCCTGTCCTTCACCATCTCC-3′, *Drd2*   reverse   5′-GACCAGCAGAGTGACGATGA-3′.

Data were interpreted using 2^−ΔCT^ and 2^−ΔΔCT^ methods (Livak and Schmittgen, 2001). To compare gene expression between age groups at Pre-extinction, the 2^−ΔCT^ method was used. To examine change in gene expression between Pre-extinction and Post-extinction groups, the 2^−ΔΔCT^ method was used. Because prefrontal neural networks are governed by a balance of D1R vs. D2R signaling (Seamans and Yang, 2004), gene expression was also analyzed as a ratio using 2^−ΔCT^D1R/D2R (ΔCT_D1R/D2R_ = CT_D1R_−CT_D2R_) and 2^−ΔΔCT^ (ΔΔCT_D1R/D2R_ = ΔCT_D1R/D2R_−(CT_D1R average Pre-extinction_ − CT_D2R average Pre-extinction_)).

### Data Analysis and Baseline Levels of Freezing

Statistical tests were conducted using SPSS (IBM Corp., New York, NY, USA), with acceptance for significance at *p* ≤ 0.05. Data were analyzed using one-way or repeated-measures analysis of variance (ANOVA; Tukey HSD multiple comparisons) appropriate to each experimental design. For analyses of within-session extinction, data were collapsed into six blocks of five CS presentations per block (Extinction blocks). Significant interactions were followed by *post hoc* per factor ANOVA (Tukey HSD multiple comparisons), or *t*-tests (when only two groups were in a factor) as described previously (Kim and Richardson, 2010; Ganella et al., 2016).

Analyses of baseline freezing for the first experiment showed no effect of age at conditioning, extinction or test. For the second experiment, there was no age effect on baseline at conditioning. There was an effect of age at extinction, however RM ANCOVA revealed that when baseline was controlled for, there was no effect of age on freezing during extinction. There was no effect of age on baseline at test. There was no effect in baseline freezing at any phase of the final experiment (*p*s > 0.05).

## Results

### Adolescents Display Extinction Deficits Compared to Adults

**Figure 1:**
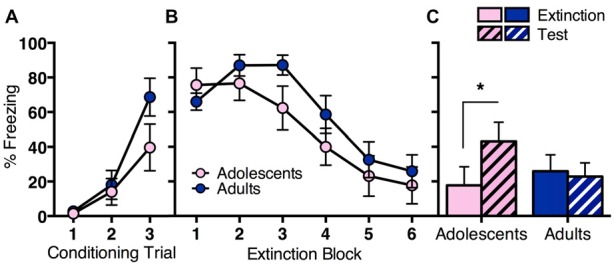
**Long-term extinction was impaired in adolescent rats compared to adult rats despite comparable within-session extinction.** Adolescents *n* = 11, adults *n* = 13. **(A)** Adolescents and adults showed comparable conditioned stimulus (CS)-elicited freezing during fear conditioning. **(B)** Adolescent and adult rats showed a similar decrease in CS-elicited freezing over extinction. **(C)** Adolescents reinstated CS-elicited freezing 24 h after extinction training, characteristic of a deficit in long-term extinction at this age. Adult rats maintained low levels of CS-elicited freezing when tested 24 h after extinction training. Data represent mean ± SEM. *p < 0.05.

The first experiment aimed to elucidate behavioral differences in fear extinction across adolescence (Figure 1). Adolescent (*n* = 11) and adult (*n* = 13) rats were conditioned with three trials of tone (CS) paired with an electric footshock (US). CS-elicited freezing during conditioning was similar across age groups (Figure 1A). RM ANOVA showed a significant effect of conditioning trial (*F*_(2,44)_ = 23.9, *p* < 0.05), with no effect of age (*F*_(1,22)_ = 2.1, *p* = 0.2) and no interaction (*F*_(6,44)_ = 1.9, *p* = 0.2). The next day, rats received extinction training consisting of 30 CS-alone trials. Both age groups showed initial high levels of freezing to the CS that decreased over the 30 CS-alone trials (Figure 1B). RM ANOVA showed a significant effect of extinction block (*F*_(5,110)_ = 30.1, *p* < 0.05), with no effect of age (*F* < 1) and no interaction (*F*_(5,110)_ = 1.6, *p* = 0.2). Adolescents reinstated CS-elicited freezing when tested the next day, whereas adults did not (Figure 1C). RM ANOVA of the final block of extinction compared to test revealed a significant overall effect of day (*F*_(1,22)_ = 6.1, *p* < 0.05), and a significant interaction between day and age (*F*_(1,22)_ = 9.9, *p* < 0.05), with no effect of age (*F* < 1). *Post hoc* tests found a significant difference in freezing levels at extinction vs. test for adolescents (*t*_(10)_ = 3.0, *p* < 0.05), but not for adults (*t* < 1). Together, these findings indicate that long-term extinction is age-related, with adolescent rats displaying impaired extinction compared to adults.

### Age Differences in mPFC Dopamine Receptor Gene Expression before and after Extinction

**Figure 2:**
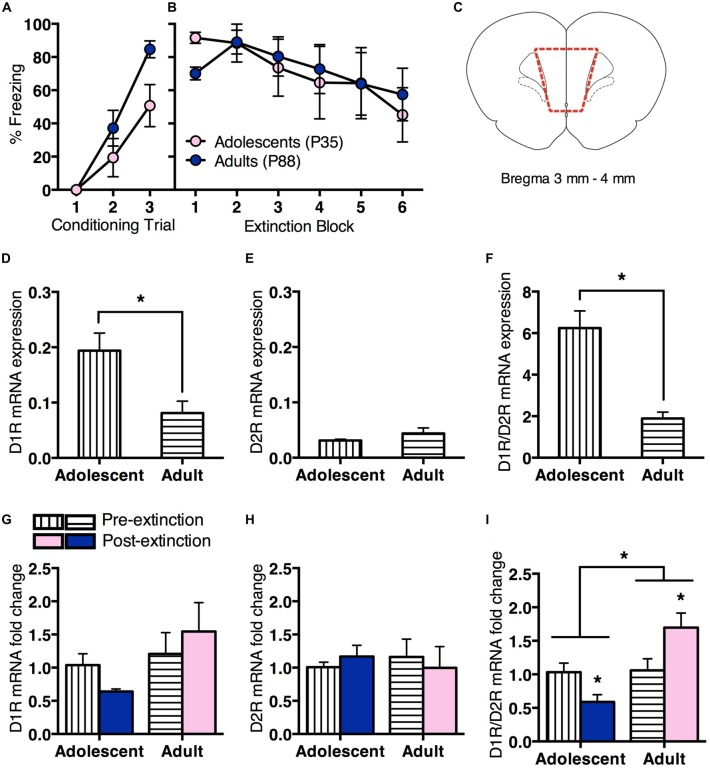
**Prefrontal dopamine receptor gene expression differed for adolescent and adult rats before and after extinction.** Adolescents *n* = 8, adults *n* = 12. **(A)** Adolescents and adults showed an increase in CS-elicited freezing over repeated pairings of the CS (tone) and the unconditioned stimulus (US; foot shock). **(B)** Adolescents and adults that received extinction training showed a similar decrease in within-session CS-elicited freezing. **(C)** Coronal section illustrating medial prefrontal cortex (mPFC) collected for quantitative PCR (qPCR) analyses. Section within broken line indicates microdissected tissue. **(D)** Pre-extinction prefrontal dopamine 1 receptor (D1R) gene expression was higher in adolescents compared to adults. **(E)** There were no differences in prefrontal dopamine 2 receptor (D2R) gene expression prior to extinction. **(F)** Pre-extinction prefrontal D1R/D2R ratio was higher in adolescents compared to adults. **(G)** There were no changes in prefrontal D1R or **(H)** D2R gene expression following extinction, however **(I)** D1R/D2R ratio was significantly downregulated in adolescents and upregulated in adults following extinction. Data represent mean ± SEM. *p < 0.05.

We then measured changes in prefrontal D1R vs. D2R gene expression pre- or post-extinction (Figure 2). Adolescent (*n* = 8) and adult (*n* = 12) rats received conditioning as described in the first experiment. All rats showed a significant increase in CS-elicited freezing during conditioning, however, the overall freezing levels were different between age groups (Figure 2A). RM ANOVA revealed an effect of conditioning trial (*F*_(2,36)_ = 34.4, *p* < 0.05) and an effect of age (*F*_(1,18)_ = 6.4, *p* = 0.02), but no interaction (*F*_(2,36)_ = 2.1, *p* = 0.1). Twenty-four hours after fear conditioning, half the rats were handled for 2 min without exposure to behavioral chambers (Pre-extinction). Remaining animals underwent extinction as per the first experiment (Post-extinction). Tissue was collected 2 h later. Adolescent and adult rats that received extinction showed comparable within-session extinction (Figure 2B). RM ANCOVA revealed that when baseline freezing was controlled for, there was an overall effect of extinction block (*F*_(5,35)_ = 3.4, *p* < 0.05), with no effect of age (*F*_(1,7)_ = 1.2, *p* = 0.3), and no effect of baseline (*F*_(1,7)_ = 2.7, *p* = 0.1). These results were the same with RM ANOVA.

Adolescents showed higher prefrontal D1R mRNA expression than adults pre-extinction (*t*_(8)_ = 3.1, *p* < 0.05; Figure 2D). There was no age difference for D2R mRNA expression (*t* < 1; Figure 2E), however adolescents displayed a significantly higher D1R/D2R ratio than adults (*t*_(8)_ = 5.7, *p* < 0.05; Figure 2F).

There was no change in D1R mRNA expression at post-extinction relative to pre-extinction for either age, with ANOVA showing no effect of treatment (*F* < 1), no effect of age (*F*_(1,16)_ = 2.4, *p* = 0.1) and no interaction (*F*_(1,16)_ = 1.1, *p* = 0.3; Figure 2G). There was also no change in D2R mRNA expression at post-extinction compared to pre-extinction for either age, with ANOVA showing no effect of treatment, age and no interaction (*F*s < 1; Figure 2H). However, there was a significant difference in D1R/D2R mRNA ratio at post-extinction relative to pre-extinction for each age. ANOVA showed a significant effect of age (*F*_(1,16)_ = 9.4, *p* < 0.05) and a significant interaction between treatment and age (*F*_(1,16)_ = 8.5, *p* < 0.05), and no effect of treatment (*F*_(1,16)_ = 0.3, *p* = 0.6). *Post hoc* tests found that adolescent D1R/D2R mRNA ratio was significantly decreased following extinction (*t*_(6)_ = 2.6, *p* < 0.05), while adult D1R/D2R mRNA ratio was significantly increased (*t*_(10)_ = 2.3, *p* < 0.05; Figure 2I). These results suggest an age difference in prefrontal D1R/D2R mRNA ratio before fear extinction, driven by increased D1R mRNA expression in adolescent rats. Notably, D1R/D2R mRNA ratio changes in the opposite direction following extinction in adolescent or adult rats.

### Enhancing IL D2R Signaling Facilitates Long-Term Extinction in Adolescents but Not Adults

**Figure 3:**
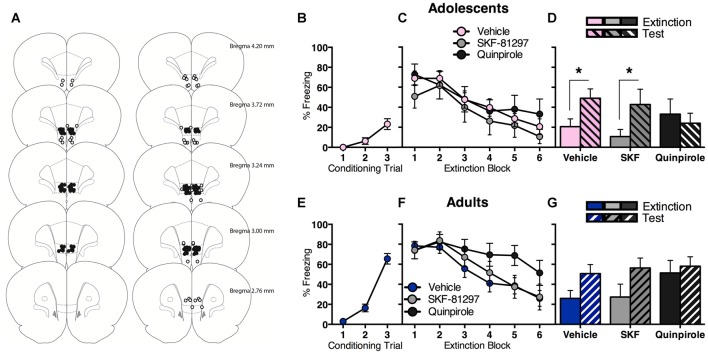
**Intra-infralimbic cortex (IL) infusions of a D1R agonist (SKF-81297) or a D2R agonist (quinpirole) had different effects on within-session and long-term extinction for adolescent and adult rats. (A)** Coronal sections illustrating intracranial cannula placements in adolescents (left) and adults (right). Bilateral cannula targeted the IL. Hits (filled circles; adolescents *n* = 36, adults *n* = 41) and misses (empty circles; adolescents *n* = 6, adults *n* = 15). **(B)** Adolescents displayed an increase in CS-elicited freezing across fear conditioning. **(C)** Acutely manipulating IL D1R or D2R signaling had no effect on adolescent extinction within-session, with all rats showing initial high levels of CS-elicited freezing that decreased as the extinction proceeded. **(D)** Adolescents that received in intra-IL vehicle or SKF-81297 at the time of extinction returned to high levels of CS-elicited freezing when tested the next day, while adolescents that received intra-IL quinpirole did not. **(E)** Adults displayed an increase in CS-elicited freezing across fear conditioning. **(F)** Acutely manipulating adult IL D2R signaling transiently impaired within-session extinction, however all adult rats inhibited CS-elicited freezing to a comparable level by the end of extinction training, irrespective of intracranial drug treatment. **(G)** Enhancing IL D1R or D2R signaling at the time of extinction training had no effect on long-term extinction in adults. Data represent mean ± SEM. *p < 0.05.

We observed that prefrontal dopamine receptor gene expression is modulated in opposite directions following fear extinction in adolescence vs. adulthood. In order to investigate potential functional implications, we administered D1R or D2R agonist into the IL of the mPFC (Figure 3A), a brain region strongly implicated in adolescent deficit of extinction (Kim et al., 2011; Pattwell et al., 2012).

Adolescents displayed a significant increase in CS-elicited freezing across conditioning (Figure 3B; *F*_(2,70)_ = 13.5, *p* < 0.05). The next day, all adolescents showed comparable extinction with no differences between drug groups (Figure 3C). RM ANOVA showed an effect of extinction block (*F*_(5,165)_ = 16.9, *p* < 0.05), with no effect of drug and no interaction (*F*s < 1). Interestingly, quinpirole prevented the return of extinguished fear at test (Figure 3D). RM ANOVA of the final block of extinction compared to test revealed an effect of day (*F*_(1,33)_ = 6.3, *p* < 0.05) and an interaction between day and drug (*F*_(2,33)_ = 3.5, *p* < 0.05), and no effect of drug (*F* < 1). *Post hoc* tests found a significant difference in freezing levels at extinction vs. test for vehicle (*t*_(18)_ = 3.6, *p* < 0.05) and SKF-81297 (*t*_(7)_ = 2.6, *p* < 0.05), but not for quinpirole (*t* < 1). Thus, acutely enhancing IL D2R signaling at the time of extinction improved long-term extinction in adolescents.

Adults also displayed a significant increase in CS-elicited freezing during conditioning (Figure 3E; *F*_(2,80)_ = 75.1, *p* < 0.05). The next day, acutely manipulating IL D1R or D2R signaling in adults had a transient effect on within-session extinction, however all adults inhibited freezing to a comparable level by the end of extinction training (Figure 3F). RM ANOVA showed an effect of extinction block (*F*_(5,190)_ = 24.9, *p* < 0.05) and a block × drug interaction (*F*_(10,190)_ = 2.0, *p* < 0.05), but no overall effect of drug (*F*_(2,38)_ = 1.6, *p* = 0.2). When the interaction was examined with one-way ANOVA of individual extinction blocks (with Tukey HSD multiple comparisons), an effect of drug at extinction block 5 only was revealed (*F*_(2,38)_ = 3.3, *p* < 0.05), with quinpirole group freezing higher than the other two groups (*p*s < 0.05). This result indicates that quinpirole delayed within-session extinction for adults. RM ANOVA of extinction vs. test showed an effect of day (*F*_(1,38)_ = 13.0, *p* < 0.05), with no effect of drug (*F*_(2,38)_ = 1.0, *p* = 0.4), and no interaction (*F*_(2,38)_ = 1.4, *p* = 0.2). Thus increasing IL D1R or D2R signaling at the time of extinction had no effect on long-term extinction in adults (Figure 3G).

## Discussion

Here we show that adolescent rats display a deficit in long-term extinction of a conditioned fear response compared to adult rats. We also showed that D1R/D2R ratio is decreased following extinction in adolescents and increased in adults. Further, enhancing IL D2R signaling using quinpirole improved long-term extinction in adolescent rats but delayed extinction acquisition in adult rats, while increasing IL D1R signaling using SKF-81297 had no effects at any age. Present findings further highlight that adolescent extinction impairments relate to developmental changes in mPFC function, and identify for the first time that maturation of PFC dopamine signaling plays a role.

Our behavioral findings are consistent with previous studies that report impaired fear extinction in adolescents compared to adults in both rodents and humans (McCallum et al., 2010; Kim et al., 2011; Pattwell et al., 2012; Baker and Richardson, 2015). These data add to a growing literature suggesting that adolescence is characterized by impairments in cue extinction more broadly, since adolescent rats also display deficits in extinction of a cocaine-associated context (Brenhouse and Andersen, 2008) and a cocaine-associated cue (Zbukvic et al., 2016). Importantly, the present findings recapitulate clinical evidence that extinction-based therapy for anxiety disorders is less effective in adolescents compared to other ages (Southam-Gerow et al., 2001; Bodden et al., 2008).

Prior to extinction, adolescents displayed increased D1R and D1R/D2R ratio mRNA compared to adults. This is consistent with studies that report a peak in D1R gene expression (Rothmond et al., 2012; Garske et al., 2013) and receptor expression (Andersen et al., 2000; Brenhouse et al., 2008) in the PFC during adolescence. In particular, previous findings indicate that early life adversity can exacerbate an adolescent peak in D1R expression on PFC projection neurons in rats (Brenhouse et al., 2013). Therefore, the increased adolescent D1R/D2R ratio observed in the present study may be a result of fear conditioning. By comparison, we found no age difference in D2R gene expression, in line with reports that D2R expression in the mPFC reaches stable adult levels by adolescence (Tarazi et al., 1998).

Since patterns of basal D1R and D2R mRNA expression in the cortex are found to correlate with receptor binding (Weiner et al., 1991), the current findings imply a markedly different prefrontal dopaminergic environment pre-extinction depending on age, with adolescent mPFC networks likely dominated by D1R activity relative to D2R activity compared to adults. Computational modeling predicts that when the PFC is dominated by D1R relative to D2R signaling, this produces a state of net inhibition (Seamans and Yang, 2004). Notably, the present findings in adolescents are similar to reports of rats with lesions of the mPFC, where fear conditioning and within-session extinction learning are intact but long-term extinction is impaired (Quirk et al., 2000; Garcia et al., 2006). Moreover, it appears that the mPFC is not recruited as efficiently during fear extinction in adolescence compared to adulthood (Kim et al., 2011; Pattwell et al., 2012; Baker and Richardson, 2015). In humans, the intense emotionality of adolescents is thought be at least partly due to an under-recruitment of the PFC (Somerville et al., 2010). The present findings suggest that the mPFC dopaminergic profile may contribute to adolescent emotionality.

Since we observed age differences in prefrontal dopamine receptor gene expression following extinction learning, we then sought to examine functional differences in dopamine signaling across adolescence vs. adulthood. Therefore, we investigated the immediate and long-term effect of enhancing D1R or D2R signaling in the IL of the PFC at the time of extinction learning. This subregion of the PFC is homologous to Brodmann Area 25 in the human brain (Gass and Chandler, 2013). Critically, both the IL of rodents and the corresponding ventromedial PFC in humans have been strongly implicated in extinction learning and retrieval, whereas the prelimbic subregion (PrL) is involved in fear expression (Quirk et al., 2000; Phelps et al., 2004; Sierra-Mercado et al., 2011). Changes in dopamine receptor gene expression following extinction, and the opposite effects observed in adolescent vs. adult rats following the manipulation of prefrontal D2R activity suggest a fundamental dissociation in dopaminergic signaling in the mPFC in relation to extinction across adolescence. Adolescent data are consistent with previous findings that intra-IL quinpirole improves extinction of a discrete cocaine-associated cue in adolescent rats (Zbukvic et al., 2016), suggesting a role for prefrontal D2R signaling across extinction learning broadly. In contrast, intra-IL quinpirole delayed the acquisition of extinction in adult rats, with no effect on long-term extinction. This is consistent with previous reports of systemic quinpirole delaying extinction in adult rats (Nader and LeDoux, 1999; Ponnusamy et al., 2005). However, systemic or ICV treatment with the D2R antagonist haloperidol (Holtzman-Assif et al., 2010), or intra-IL or systemic treatment with the D2R antagonist raclopride (Mueller et al., 2010) at the time of extinction have been shown to impair the retrieval of extinction the next day in adult rats. Disparities between findings may be due to the specificity of agonists and antagonists used, and/or route of administration (Gehlert et al., 1992; Bowery et al., 1994; Tseng and O’Donnell, 2007). Present findings suggest that the level of D2R signaling in the adult IL may be naturally optimal compared to adolescents for extinction learning, and further activation by quinpirole might disrupt this balance. Overall, divergent effects of intra-IL quinpirole add to gene expression data suggesting that that the IL of the PFC is involved in extinction learning during adolescence as well as adulthood, however that extinction may involve differential D2R signaling across development. In contrast, we found the D1R agonist SKF-81297 had no effects on within-session or long-term extinction in either adolescent or adult rats. Previous studies using adult rodents report that attenuating IL D1R signaling impairs extinction (Hikind and Maroun, 2008; Fricks-Gleason et al., 2012). By comparison, systemic treatment with a D1R agonist enhanced extinction of cued and contextual fear in adult rats, though the anatomical targets of that effect were not clear (Abraham et al., 2016). It may be that enhancing IL D1R activity is not sufficient for extinction in adolescence and adulthood.

We note that in the final experiment of the present study, we observed a small spontaneous recovery of extinguished freezing 24 h following extinction in adult rats that received saline infusion into the IL before extinction. This was not observed in our first experiment, as well as in our and other groups’ previous studies (Quirk, 2002; McCallum et al., 2010; Kim et al., 2011; Orsini et al., 2011). A careful examination of the literature revealed that pre-extinction infusion of saline or vehicle into the IL or the PrL, but not other brain regions, may cause this effect in adult rats (Sierra-Mercado et al., 2011). This small but significant spontaneous recovery due to pre-extinction vehicle infusion appears to also be present even when the freezing was well extinguished to baseline (i.e., ~0%), and when vehicle has also been infused during test to provide identical physiological contexts for extinction and test (Laurent and Westbrook, 2008). In studies that do not involves such saline or vehicle infusions into the IL, adult rodents maintain low levels of freezing following extinction (Quirk, 2002; McCallum et al., 2010; Kim et al., 2011; Orsini et al., 2011). Therefore, we believe that the result of our final experiment does not affect the interpretation of our data. Additionally, there was a significant effect of age during conditioning for qPCR experiment, whereas there was only a trend in the behavioral experiment. Based on the results of microinfusion experiments, we believe that adolescent rats showed reduced freezing during conditioning compared to adults in the present study. While these results are inconsistent with previous findings (McCallum et al., 2010; Kim et al., 2011), there are more recent studies that do not report conditioning data in extinction across adolescence (Pattwell et al., 2012, 2016). Importantly, freezing levels during extinction were comparable in adolescent and adults in all experiments of the present study.

Further studies are required to confirm age differences in D1R vs. D2R protein expression following fear conditioning and extinction, although antibodies that are specific to D1R or D2R, and not any other dopamine receptor subtypes are notoriously lacking. Specifically, D1R and D2R proteins show striking structural similarities, which have implications for visualization and quantification in brain tissue. For instance, sequence similarity searching using the Basic Local Alignment Search Tool (BLAST) database reveals that D1R and D2R share 77% of their amino acid sequence (Agostino, 2012). It follows that D1R and D2R display similar ligand binding profiles (Levey et al., 1993). This means that commercially available antibodies for D1R and D2R are liable to display cross-reactivity (Michel et al., 2009; Hutchings et al., 2010). Immunostaining of D2R in particular has historically shown conflicting results across previous literature, with some studies reporting extensive labeling throughout all layers of cortex (Ariano et al., 1993), while others have shown little to no staining (Levey et al., 1993; Sesack et al., 1994). Therefore, present qPCR findings offer an exciting first step to elucidating how the maturing prefrontal dopamine system may contribute to extinction across adolescence.

Epidemiological data indicate that persistence of mental health problems among adolescents relates more to recurrence rather than chronicity of youth-onset disorders (Kessler et al., 2012). Our data suggest that this may be due, at least in part, to extinction impairments at this age. Not only do we demonstrate that adolescent rats are impaired in long-term extinction, we also show for the first time that extinction produces unique changes dopamine receptor gene expression across development. Importantly, present findings highlight D2R as a promising pharmacological target to improve exposure therapy in adolescents, which is consistent with our recent observation that the partial agonist of the D2R, aripiprazole, can significantly facilitate long-term extinction in adolescent rats (Ganella et al. under review). While behavioral therapies that involve exposure therapy are the most effective way to treat anxiety disorders, less than one in five adolescents have received therapy for their anxiety (Merikangas et al., 2011). We propose that an effective pharmacological adjunct that acutely accompanies behavioral therapy could significantly reduce the amount of treatment necessary during this vulnerable period, and reduce chronic use of medication. Given that neural correlates of adolescent behavior are highly conserved across species (Spear, 2000), present findings represent an important step to developing more effective treatments for adolescents living with anxiety disorders.

## Author Contributions

JHK and ICZ conceptualized and designed the study. ICZ, CHJP and DEG acquired the data. ICZ and JHK analyzed the data. ICZ, AJL and JHK interpreted the data. All authors were involved in critically revising the work for important intellectual content and in final approval of the version to be published. All authors agree to be accountable for all aspects of the work in ensuring that questions related to the accuracy or integrity of any part of the work are appropriately investigated and resolved.

## Funding

This work was supported by an Australian Postgraduate Award awarded to ICZ, Baker Foundation Fellowship awarded to DEG, a Principal Research Fellowship (APP1020737) from the National Health and Medical Research Council (NHMRC) of Australia awarded to AJL, NHMRC Career Development Fellowship (grant number APP1083309) awarded to JHK and NHMRC Project grant (APP1063140) awarded to JHK and AJL. Australian Research Council Discovery Grant (DP150102496) awarded to JHK.

## Conflict of Interest Statement

The authors declare that the research was conducted in the absence of any commercial or financial relationships that could be construed as a potential conflict of interest.
